# DNMT1-targeted macrophages delivering drug-loaded nanoparticles attenuate heart allograft fibrosis via PTPRD-STAT3/6 axis

**DOI:** 10.1038/s41420-026-03295-5

**Published:** 2026-08-07

**Authors:** Naonao Yuan, Zhibo Ma, Zihan Xue, Qingwen Li, Xi Zhou, Jing Liu, Yanping Lin, Shiqi Gu, Sichuan Yi, Yao Lu, Peixiang Lan

**Affiliations:** 1 Institute of Organ Transplantation, Tongji Hospital, Tongji Medical College, Huazhong University of Science and Technology, Key Laboratory of Organ Transplantation, Ministry of Education, NHC Key Laboratory of Organ Transplantation, Key Laboratory of Organ Transplantation, Chinese Academy of Medical Sciences, Organ Transplantation Clinical Medical Research Center of Hubei Province, Wuhan, China; 2 School of Pharmacy, Tongji Medical Colloge, Huazhong University of Science and Technology, Wuhan, China; 3 Department of Nephrology, Tongji Hospital, Tongji Medical College, Huazhong University of Science and Technology, Wuhan, China

**Keywords:** Immunology, Diseases, Immunotherapy, Cardiovascular diseases

## Abstract

Distinct macrophage activation states play key roles in the formation and progression of heart allograft fibrosis. However, the exact molecular mechanisms that regulate the balance between inflammatory and pro-fibrotic macrophage responses during fibrosis remain poorly understood. Identifying and targeting the key molecule that regulates the differentiation of inflammatory and pro-fibrotic macrophages might be a pivotal strategy for mitigating fibrosis and prolonging allograft survival. Here, using single-cell sequencing analysis of human heart allografts fibrosis patient samples, we identified a population of macrophages that undergo epigenetic reprogramming through DNA methylation regulated by DNA methyltransferase DNMT1. DNMT1 small-molecule inhibitor 5-AzaC significantly attenuated graft fibrosis and prolonged allograft survival by inhibiting both inflammation and excessive tissue repair. Mechanistically, DNMT1-targeted macrophages suppress the DNA methylation level of the CpG island in the first intron of PTPRD, promoting PTPRD expression, which in turn inhibits the activity of STAT3 and STAT6 pathways, thereby suppressing the differentiation of inflammation and pro-fibrotic macrophages. Based on this, we developed a macrophage-targeted nanocarrier, MV/PEG-LPs, to load 5-AzaC, thereby enhancing its therapeutic precision for allograft fibrosis. Our results reveal a novel target, DNA methylation in macrophages, for the treatment of fibrosis-related diseases.

**Figure:**
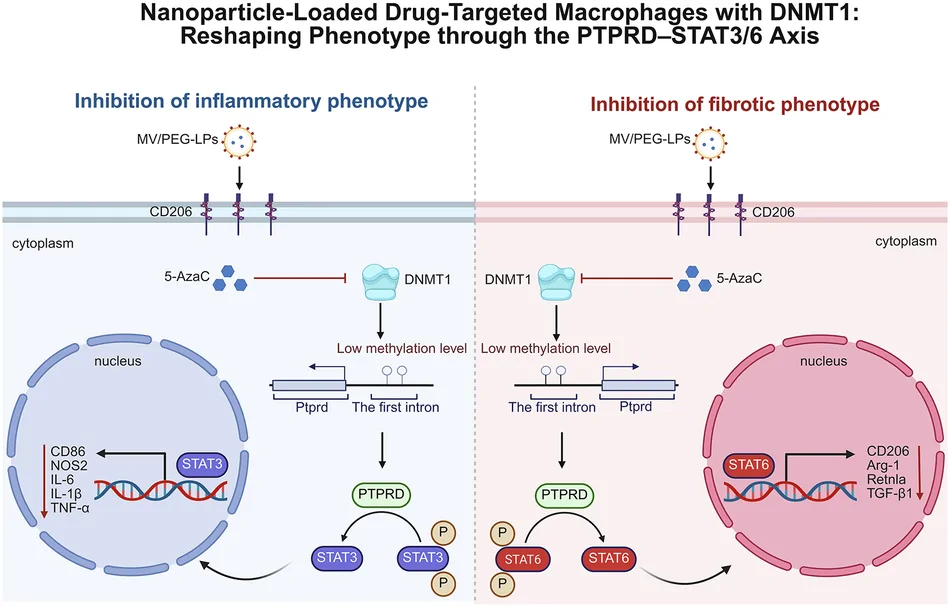

## Introduction

Tissue fibrosis, characterized by the excessive accumulation of extracellular matrix (ECM) components, is a highly dynamic and complex process that represents one of the leading causes of disability and mortality worldwide [1, 2]. Fibrosis also contributes to chronic allograft rejection, ultimately leading to graft dysfunction [3]. Although the pathological conditions that initiate and sustain tissue fibrosis are diverse, the terminal stages of fibrosis appear remarkably stereotyped from biochemical and histopathological perspectives [4]. Specifically, the early stages of fibrosis are marked by immune-inflammatory features, including the infiltration of monocytes around blood vessels and an imbalance in the spectrum of anti-fibrotic and pro-fibrotic cytokines [5]. Therefore, accurately recognizing the complexity of fibrosis progression and identifying targets that simultaneously address the early inflammatory responses and the late imbalance in pro-fibrotic factor secretion are of paramount importance [6, 7].

Macrophages are critical effector cells in both the progression and resolution of fibrosis [8]. During the early stages of organ transplantation, ischemia-reperfusion injury releases a plethora of damage-associated molecular patterns (DAMPs), inflammatory cytokines, chemokines, metabolites, and membrane protein ligands from donor cells [9]. These factors can recruit and activate monocyte-derived macrophages through various pathways, promoting their differentiation into pro-inflammatory M1 macrophages [10]. M1 macrophages produce inflammatory chemokines and high levels of pro-inflammatory cytokines, such as CXCL9, CXCL10, CXCL16, TNF, IL-1β, IL-6, IL-12, and IL-23 [11]. They also activate adaptive immune responses by upregulating co-stimulatory molecules and major histocompatibility complexes, including CD40, CD80, CD86, and MHC II, thereby exacerbating graft inflammation and tissue cell death [12, 13]. As transplantation progresses, particularly during chronic rejection phases, macrophages often undergo transcriptional reprogramming, shifting towards an alternatively activated phenotype (M2 type) [14]. M2 macrophages are generally considered to have anti-inflammatory and immunoregulatory functions, expressing molecules such as TGF-β, IL-4, IL-13, and vascular endothelial growth factor (VEGF) [15, 16], which are involved in suppressing inflammation and promoting tissue repair. However, excessive repair by M2 macrophages can accelerate interstitial fibrosis in the graft, ultimately leading to organ dysfunction [17, 18].

Traditional immunosuppressants, which inhibit post-transplant immune responses to maintain graft function, do not appear to benefit long-term graft survival and are associated with a range of side effects, including tissue fibrosis, graft dysfunction, and cardiovascular diseases [19, 20]. Targeting fibrosis could be a strategic approach to prolong allograft survival. Unfortunately, drugs aimed at targeting end-stage fibrosis have not yet been translated into clinical treatments. In this study, we utilized single-cell sequencing to identify a population of macrophages in fibrotic grafts that undergo DNA methylation epigenetic reprogramming, with DNMT1 as the core driving factor. Application of the small-molecule inhibitor 5-AzaC targeting DNMT1 significantly attenuated graft fibrosis and prolonged allograft survival by simultaneously inhibiting pre-transplant inflammation and post-transplant excessive tissue repair. Based on this, we developed a macrophage-targeted nanocarrier, MV/PEG-LPs, to load 5-AzaC, thereby enhancing its therapeutic precision for allograft fibrosis. Our research demonstrates that targeting DNMT1 in macrophages could serve as a potential therapeutic target for fibrosis-related diseases.

## Results

### DNMT1^+^ macrophages are enriched in human and mouse fibrotic hearts

**Fig. 1:**
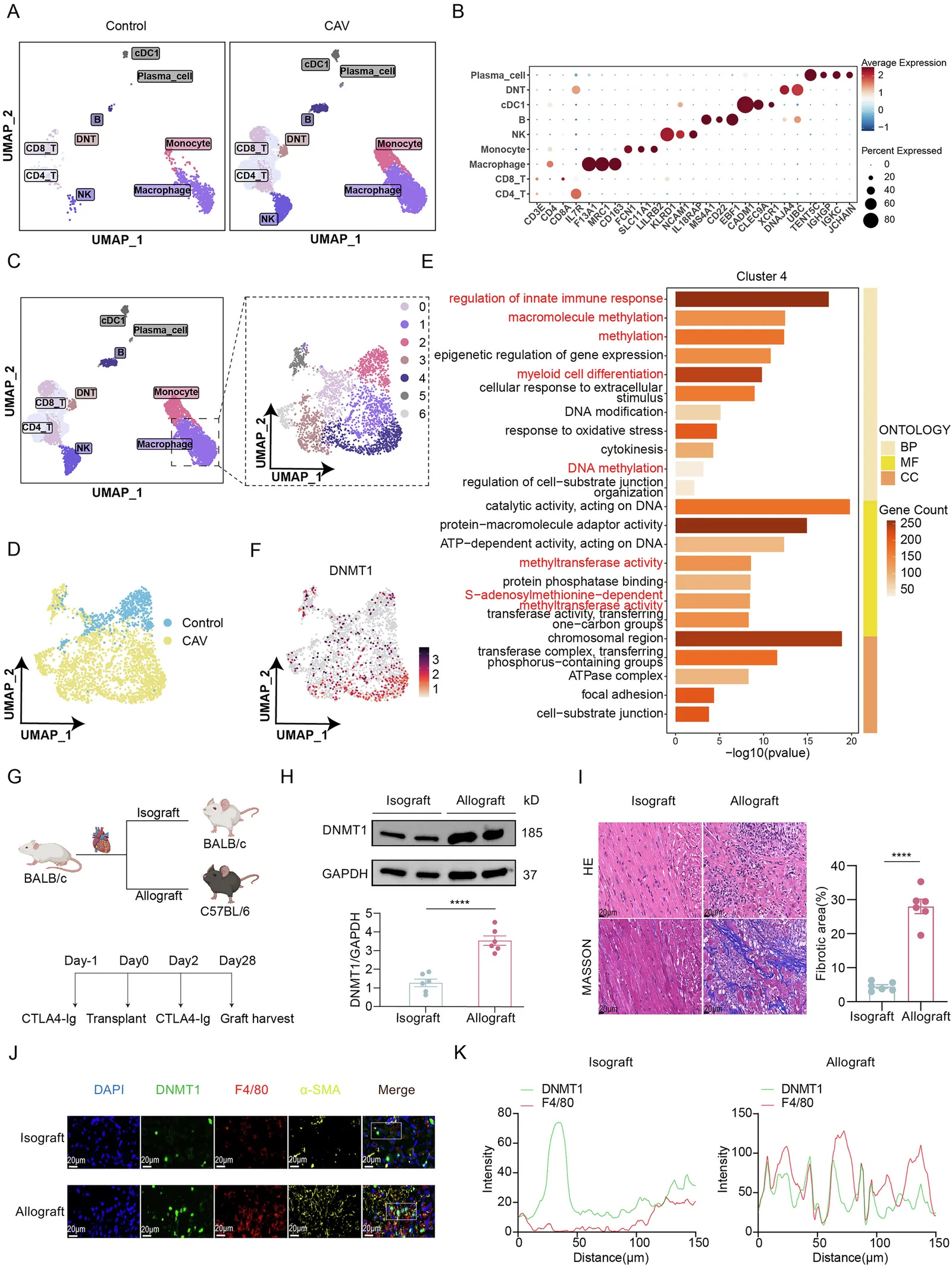
DNMT1^+^ macrophages are enriched in human and mouse fibrotic hearts. **A** UMAP plot showing distinct single-cell clusters in the immune cells of human cardiac tissue from control and CAV groups. **B** Cell markers used for clustering analysis. **C** UMAP plot of macrophage subclusters from human cardiac tissue. **D** UMAP plot of macrophage subclusters from control and CAV human cardiac tissue. **E** Top GO-enriched gene sets in cluster 4. **F** DNMT1 expression in macrophages of human cardiac tissue. **G** Construction of isograft and allograft models. **H** Western blot analysis showing DNMT1 expression in macrophages from the isograft and fibrotic allograft mouse hearts, with GAPDH used as the internal control protein. The lower panel shows the quantitative analysis of DNMT1 expression (*n* = 6). Unpaired Student’s *t* test. ****P < 0.0001. **I** Representative histological images of cardiac grafts collected on day 28 after transplantation, stained with H&E and Masson’s trichrome (scale bar:20 µm). Quantitative analysis results of the fibrotic region on the right side (*n* = 6). Unpaired Student’s *t* test. ****P < 0.0001. **J** Immunofluorescence co-localization demonstrating DNMT1 expression in cardiac macrophages of mice (scale bar: 20 μm). **K** Immunofluorescence co-localization analysis of DNMT1 and F4/80 in the rectangular regions of the isograft and allograft heart groups shown in (**J**).

We analyzed a publicly available human cardiac single-cell sequencing dataset that include 4 allograft hearts with fibrosis (cardiac allograft vasculopathy, CAV) and 3 control hearts [21] (Fig. 1A). We clustered the single cells from the CAV and control hearts based on known cell markers and identified major immune cell types (Fig. 1B), including macrophages, monocytes, CD4^+^ T cells, CD8^+^ T cells, double-negative T cells (DNT), B cells, plasma cells, cDC1, and NK cells (Fig. 1A, B). Compared with control hearts, the macrophages in the fibrotic allograft hearts showed significant differential expression characteristics (Fig. 1C). Herein, we observed prominent immune cell infiltration within fibrotic cardiac allografts (Fig. 1A). Given the critical role of macrophages in driving graft fibrosis [22], we subsequently performed unsupervised dimensionality reduction analyses to characterize macrophage subsets, which identified seven distinct populations (Fig. 1C). We observed a significant enrichment of macrophages in the CAV group (Fig. 1D). Through Gene Ontology (GO) enrichment analysis, macrophage cluster 4 was found to exhibit significant upregulation of multiple gene sets associated with epigenetic regulation, including pathways involved in DNA methylation (Fig. 1E). Notably, the expression of DNMT1 in the macrophages of the CAV group was significantly higher than that in the control group (Fig. 1F and Fig. S1A–C).

Given our previous results of a population of DNMT1^+^ macrophages enriched in human heart allografts with fibrosis, we established a murine model of chronic cardiac allograft rejection characterized by tissue fibrosis (Fig. 1G). We then collected grafts from the isograft group and fibrosis allografts at 4 weeks post-transplantation, and extracted infiltrating macrophages from both groups. Our results revealed a significant increase in DNMT1 protein levels in the fibrosis heart allografts (Fig. 1H). Meanwhile, HE and Masson staining revealed that allografts exhibited more immune cell infiltration and fibrosis compared to the isograft control group (Fig. 1I). Multiplex immunofluorescence staining results indicated that, compared to the isograft group, fibrosis heart allografts exhibited a marked increase in macrophage infiltration, elevated Dnmt1 expression, and a significant co-localization of macrophages and Dnmt1 (Fig. 1J, K). These results suggest the presence of a population of Dnmt1^+^ macrophages in fibrotic grafts, which may contribute to the progression of heart allograft fibrosis.

### DNMT1 inhibitor 5-AzaC prolonged graft survival and attenuated graft fibrosis

**Fig. 2:**
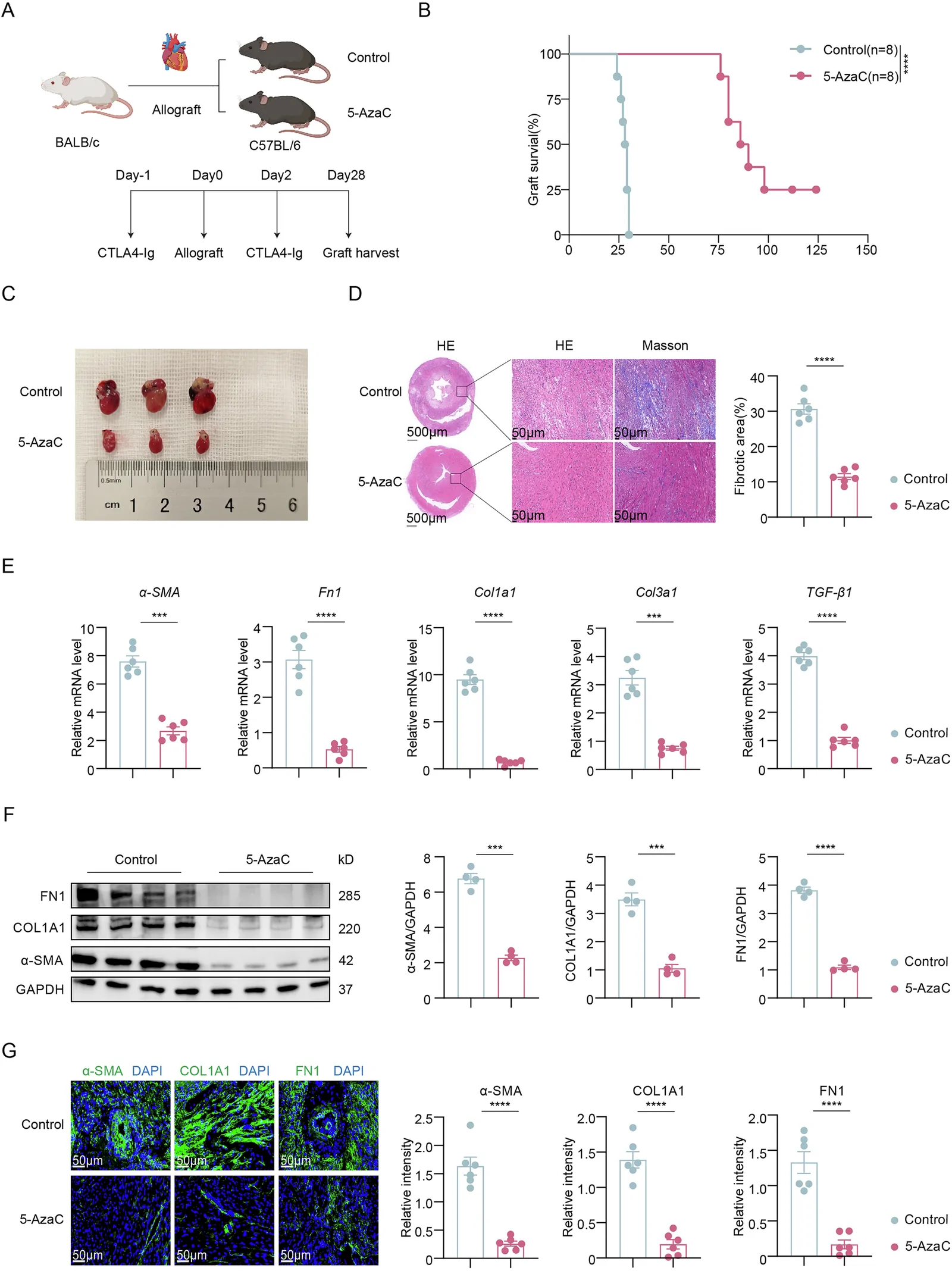
DNMT1 inhibitor 5-AzaC prolonged graft survival and attenuated graft fibrosis. **A** BALB/c heart was transplanted into recipient mice treated with CTLA4-Ig (control group) or 5-AzaC to establish an intra-abdominal heterotopic heart transplantation model. **B** The percentage of allograft survival after transplantation (*n* = 8). Log-rank (Mantel–Cox) test. ****P < 0.0001. **C** Allografts harvested on day 28 after transplantation. Representative images show the grafts from the control and 5-azacytidine-treated groups. **D** Representative histological images of cardiac allografts collected on day 28 after transplantation from the control and 5-azacytidine-treated mice, stained with H&E (scale bar: 500 or 50 µm) and Masson’s trichrome (scale bar: 50 µm). Quantitative analysis results of the fibrotic region on the right side (*n* = 6). Unpaired Student’s *t* test. ****P < 0.0001. **E** mRNA expression levels of fibrosis-related genes in cardiac allografts from control and 5-AzaC -treated mice (*n* = 6) on day 28 post-transplantation. Unpaired Student’s *t* test. ***P < 0.001; ****P < 0.0001. **F** Western blot analysis showing the expression of FN1, COL1A1, and α-SMA in grafts on day 28 after transplantation, with GAPDH used as the internal control. The quantitative analysis of FN1, COL1A1, and α-SMA expression is shown on the right (*n* = 4). Unpaired Student’s *t* test. ***P < 0.001; ****P < 0.0001. **G** Immunofluorescence staining of allograft sections on day 28 after transplantation in control and 5-azacytidine-treated mice (scale bar: 50 µm), showing FN1, COL1A1, and α-SMA (green) and DAPI staining (blue). Quantification of FN1, COL1A1, and α-SMA expression is presented on the right (*n* = 6). Unpaired Student’s *t* test. ***P < 0.001; ****P < 0.0001.

To analyze this, using the chronic rejection model (Fig. 2A), we found that mice treated with 5-AzaC (a Dnmt1 inhibitor) retained BALB/c heart allografts for a longer duration compared to PBS-treated controls (Fig. 2B). Compared to control mice, the heart allografts in 5-AzaC-treated mice were significantly smaller in volume, indicating markedly reduced levels of fibrosis and immune cell infiltration in the intervention group **(**Fig. 2C**)**. Hematoxylin and Eosin (HE) and Masson’s trichrome staining analyses revealed that, compared to control heart allografts, the 5-AzaC-treated allografts exhibited only minimal fibrosis and vasculopathy (Fig. 2D). In addition, we measured the mRNA expression levels of *α-SMA*, *Fn1*, *Col1a1*, *Col3a1* and *TGF-β1* by quantitative real-time PCR (qPCR) (Fig. 2E). Western blotting and immunofluorescence analyses were used to assess the expression of α-SMA, Col1a1, and Fn1. The expression levels of α-SMA, Col1a1, and Fn1 were significantly downregulated in 5-AzaC–treated mice (Fig. 2F, G). Thus, targeting DNMT1 suppresses fibrosis in chronic allograft rejection.

### The DNMT1 inhibitor 5-AzaC suppresses the differentiation of inflammatory and pro-fibrotic phenotype macrophages

**Fig. 3:**
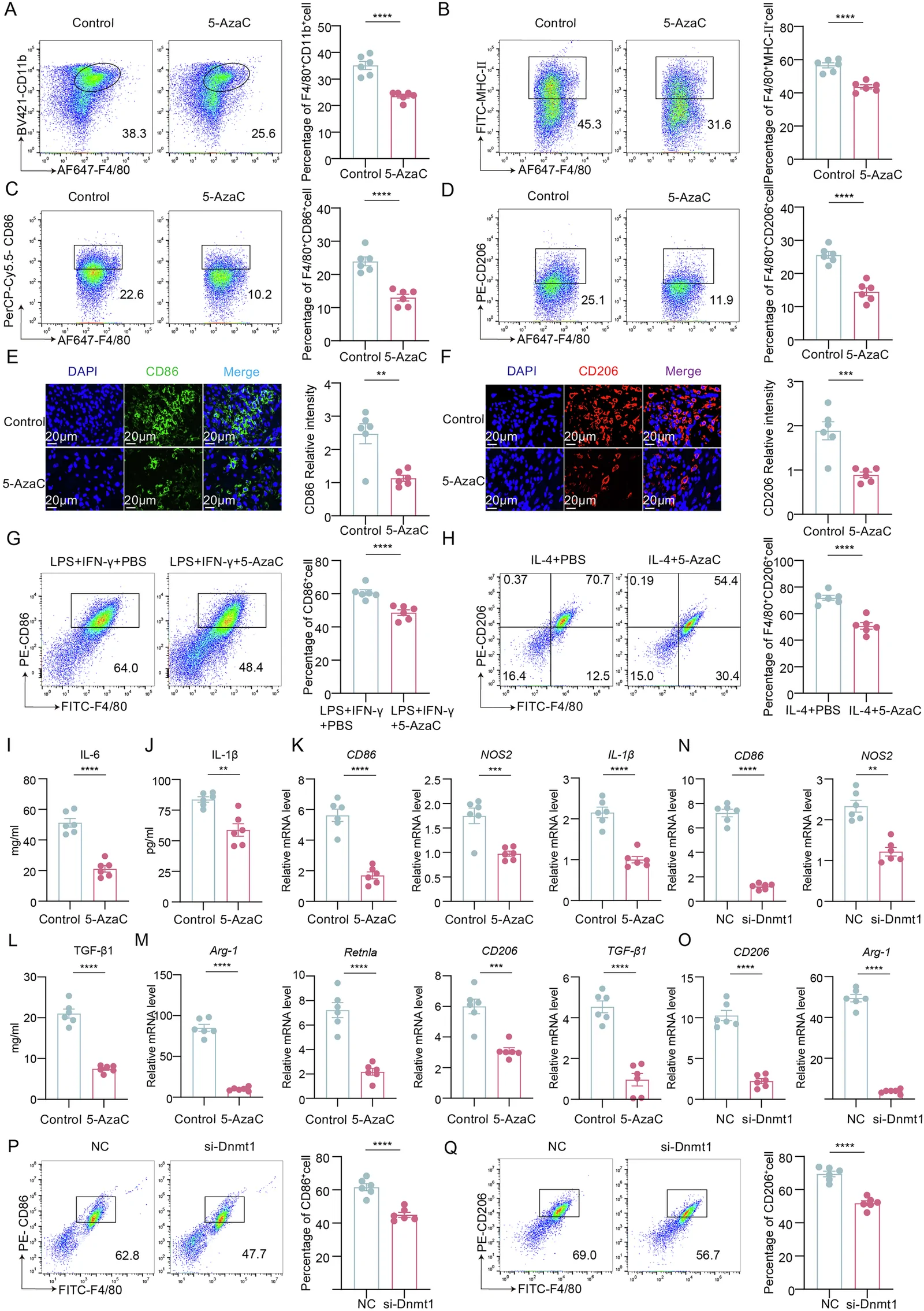
The DNMT1 inhibitor 5-AzaC suppresses the differentiation of inflammatory and pro-fibrotic phenotype macrophages. **A**–**D** Flow cytometric analysis and quantification of total macrophages, MHC-II⁺ macrophages, CD86⁺ macrophages and CD206⁺ macrophages in cardiac allografts (*n* = 6). Unpaired Student’s *t* test. ****P < 0.0001. **E** Immunofluorescence staining of CD86 (green) and DAPI (blue) in cardiac allograft sections (scale bar: 20 µm), with quantitative analysis shown on the right (*n* = 6). Unpaired Student’s *t* test. **P < 0.01. **F** Immunofluorescence staining of CD206 (red) and DAPI (blue) in cardiac allograft sections (scale bar: 20 µm), with quantitative analysis shown on the right (*n* = 6). Unpaired Student’s *t* test. ***P < 0.001. **G** Flow cytometric analysis and quantification of CD86 expression in LPS- and IFN-γ-stimulated bone marrow–derived macrophages (BMDMs) (*n* = 6). Unpaired Student’s *t* test. ****P < 0.0001. **H** Flow cytometric analysis and quantification of CD206 expression in IL‑4–stimulated bone marrow–derived macrophages (*n* = 6). Unpaired Student’s *t* test. ****P < 0.0001. **I** ELISA results showing IL-6 levels in the culture medium of LPS and IFN-γ–stimulated BMDMs. (*n* = 6). Unpaired Student’s *t* test. ****P < 0.0001. **J** ELISA results showing IL-1β levels in the culture medium of LPS and IFN-γ–stimulated BMDMs (*n* = 6). Unpaired Student’s *t* test. **P < 0.01. **K** mRNA expression levels of *CD86*, *NOS2*, and *IL‑1β* in LPS- and IFN-γ–stimulated BMDMs (*n* = 6). Unpaired Student’s *t* test. ***P < 0.001; ****P < 0.0001. **L** ELISA results showing TGF‑β1 levels in the culture medium of IL‑4–stimulated BMDMs (*n* = 6). Unpaired Student’s *t* test. ****P < 0.0001. **M** mRNA expression levels of *Arg‑1*, *Retnla*, *CD206* and *TGF‑β1* in IL‑4–stimulated BMDMs (*n* = 6). Unpaired Student’s *t* test. ***P < 0.001; ****P < 0.0001. **N** mRNA expression levels of *CD86* and *NOS2* in BMDMs transfected with siRNA-Dnmt1 followed by LPS and IFN-γ stimulation (*n* = 6). Unpaired Student’s *t* test. **P < 0.01; ****P < 0.0001. **O** mRNA expression levels of *Arg‑1* and *CD206* in BMDMs transfected with siRNA-Dnmt1 followed by IL-4 stimulation (*n* = 6). Unpaired Student’s *t* test. ****P < 0.0001. **P** Flow cytometric analysis and quantification of CD86 expression in BMDMs transfected with siRNA-Dnmt1 followed by LPS and IFN-γ stimulation. (*n* = 6). Unpaired Student’s *t* test. ****P < 0.0001. **Q** Flow cytometric analysis and quantification of CD206 expression in BMDMs transfected with siRNA-Dnmt1 followed by IL-4 stimulation (*n* = 6). Unpaired Student’s *t* test. ****P < 0.0001.

Since chronic rejection involves immune responses, we next examined the changes in immune cell infiltration in heart allografts following 5-AzaC treatment (Fig. S2A). The results showed that in the 5-AzaC-treated group, both the proportion and number of macrophages in the heart allografts were significantly reduced (Fig. 3A). In addition, the proportion and quantity of MHCII^+^ macrophages and CD86^+^ macrophages that promote inflammation were reduced (Fig. 3B, C). Meanwhile, the CD206^+^ macrophages that promote tissue fibrosis were also significantly decreased (Fig. 3D). Immunofluorescence results also indicated a significant reduction in the number of macrophages with inflammatory and pro-fibrotic phenotypes, suggesting that 5-AzaC can inhibit the differentiation of inflammatory and pro-fibrotic macrophages (Fig. 3E, F). However, the proportion and cytotoxic function of T cells in the heart allografts of 5-AzaC-treated mice did not show significant changes, indicating that the target of 5-AzaC action is macrophages (Fig. S2B).

Next, we isolated bone marrow-derived macrophages (BMDMs) in vitro and cultured them with M-CSF for 6 days to differentiate into M0-like macrophages. Subsequently, we induced M1 macrophages associated with inflammation using LPS and IFN-γ, and M2 macrophages associated with pro-fibrosis using IL-4. The results similarly demonstrated that 5-AzaC intervention significantly inhibited the differentiation of both inflammatory and pro-fibrotic phenotype macrophages. (Fig. 3G, H). Subsequently, we collected BMDM cells from the control and 5-AzaC intervention groups for ELISA analysis. The results indicated a significant reduction in the secretion of inflammatory cytokines IL-6 and IL-1β, as well as the pro-fibrotic cytokine TGF-β1(Fig. 3I–J, L). qPCR analysis of inflammatory and pro-fibrotic markers in both groups of cells further confirmed that targeting Dnmt1 in macrophages simultaneously suppresses the differentiation of both inflammatory and pro-fibrotic macrophages (Fig. 3K, M). To further validate DNMT1 as a target in macrophages, BMDMs were transfected with siRNA-Dnmt1 to assess phenotypic alterations. Our results indicate that targeting DNMT1 significantly suppresses macrophage differentiation into pro-inflammatory and pro-fibrotic phenotypes (Fig. 3N–Q).

### 5-AzaC attenuates inflammation and pro-fibrotic macrophage differentiation through the Ptprd–Stat3/6 axis

**Fig. 4:**
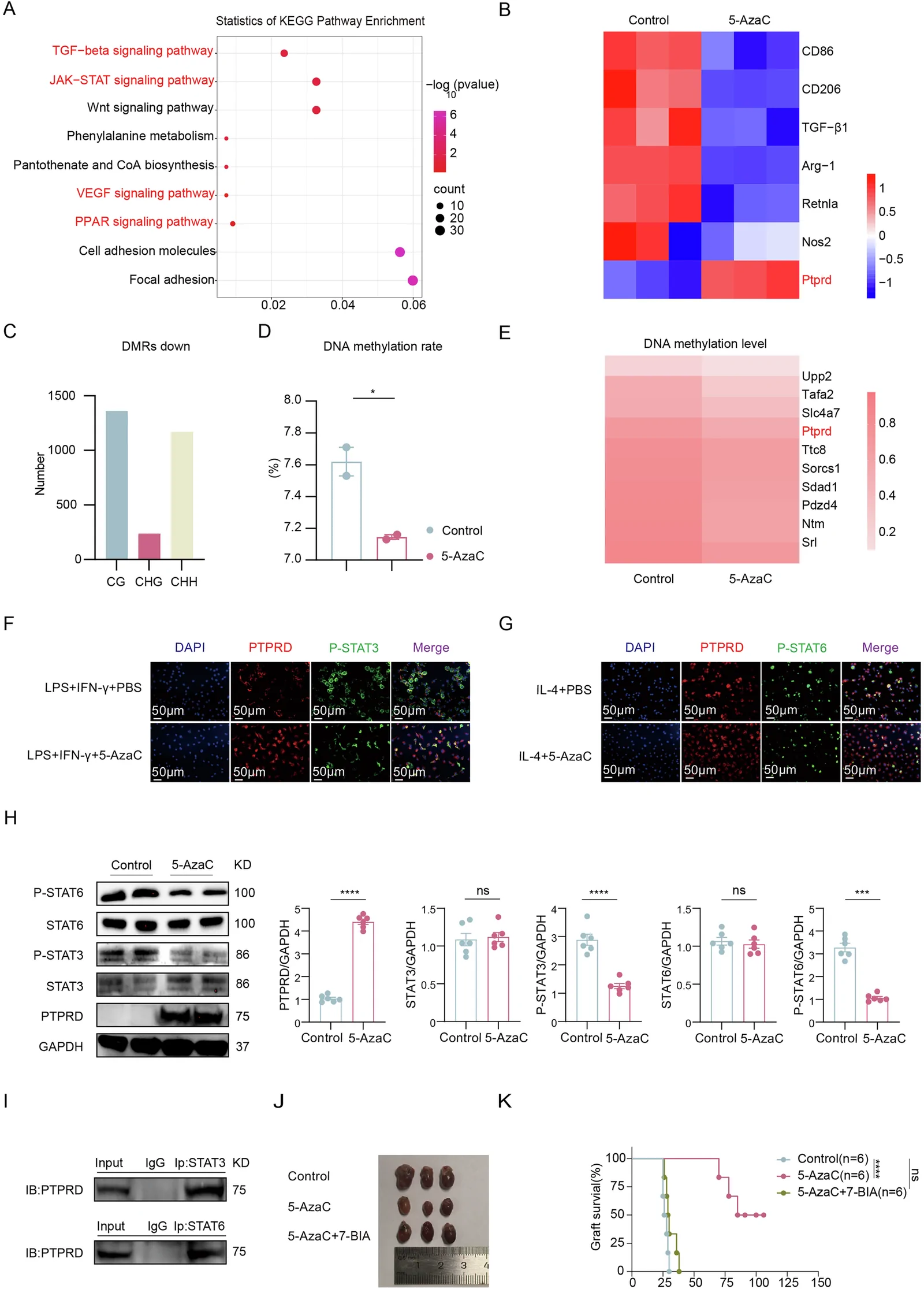
5-AzaC attenuates inflammation and pro-fibrotic macrophage differentiation through the Ptprd–Stat3/6 axis. **A** RNA sequencing analysis of macrophages from cardiac grafts at day 28 post‑transplant in control and 5‑AzaC‑treated groups. Bubble plot showing KEGG pathway enrichment analysis. **B** Heatmap displaying mRNA expression levels of *CD86*, *CD206*, *TGF-β*, *Arg-1*, *Retnla*, *Nos2* and *Ptprd* in macrophages from cardiac grafts at day 28 post‑transplant in control and 5‑AzaC‑treated groups. **C** Numbers of CG, CHG, and CHH sites in hypomethylated DMRs in control vs. 5-AzaC–treated group. **D** Genome-wide methylation levels based on WGBS analysis of macrophages from cardiac grafts at day 28 post‑transplant in control and 5‑AzaC‑treated groups. **E** Methylation levels in the first intron regions of representative genes with significant differentially methylated regions (DMRs) in the control vs. 5-AzaC–treated group. **F** Colocalization of PTPRD and P-STAT3 in LPS- and IFN-γ–stimulated BMDMs treated with or without 5‑AzaC (scale bar: 50 µm). **G** Colocalization of PTPRD and P-STAT6 in IL-4–stimulated BMDMs treated with or without 5‑AzaC (scale bar: 50 µm). **H** Western blot analysis showing PTPRD, STAT3, P-STAT3, STAT6 and P-STAT6 expression in macrophages isolated from cardiac allografts on day 28 post-transplantation, with GAPDH as the internal control. The right panel shows quantification of PTPRD, STAT3, P-STAT3, STAT6 and P-STAT6 expression (*n* = 6). Unpaired Student’s *t* test. ***P < 0.001; ****P < 0.0001; ns = not significant. **I** Immunoprecipitation using anti-STAT3 and anti-STAT6 antibodies followed by immunoblotting with an anti-PTPRD antibody. **J** Representative images of allografts harvested on day 28 after transplantation from control, 5-AzaC -treated, and 5-AzaC + 7-BIA-treated mice. **K** The percentage of allograft survival after transplantation (*n* = 6). Log-rank (Mantel–Cox) test. ****P < 0.0001.

To better identify the impact of 5-AzaC on gene expression in macrophages within heart allografts, we collected heart allografts from control and 5-AzaC treatment groups at day 28 post-transplantation and used flow cytometry to sort CD11b^+^ F4/80^+^ macrophages for transcriptome sequencing analysis (Fig. 4A). Meanwhile, we measured the expression levels of *CD86*, *CD206*, *TGF-β1*, *Arg-1*, *Retnla*, and *Nos2* in these macrophages at the mRNA level (Fig. 4B). KEGG pathway enrichment analysis revealed that the JAK-STAT pathway (Fig. 4A), which is associated with both inflammation and tissue repair [23], was significantly enriched in the infiltrating macrophages of heart allografts in the 5-AzaC treatment group, suggesting it as a potential downstream signaling pathway of DNMT1 intervention. DNA methylation is an important epigenetic mechanism for regulating cellular functional differentiation, and it frequently occurs at the CPG sites within the DNA sequence. Given the significant association of DNMT1 with DNA methylation, we next performed Whole-genome bisulfite sequencing (WGBS) to analyze the differential levels of DNA methylation in macrophages from the heart allografts of both groups following 5-AzaC intervention (Fig. 4C–E). The CPG site, also known as the CPG island. In this WGBS sequencing, we classified the CPG sites into three types: CG, CHG, and CHH. We separately examined the number of these three CPG islands in the differentially methylated regions of the two groups of samples and found that the CG type was the most significant (Fig. 4C). The results also indicated a significant reduction in DNA methylation levels in macrophages from the 5-AzaC intervention group (Fig. 4D). The heatmap analysis revealed that there were differences in the CpG islands within the first intron region of some genes, where we observed a marked decrease in the DNA methylation level of PTPRD (Fig. 4E), a potential negative regulator of the JAK-STAT pathway. The level of DNA methylation is negatively correlated with the level of gene expression [24, 25]; qPCR results indicated a significant increase in the mRNA expression level of PTPRD (Fig. 4B). PTPRD is a protein tyrosine phosphatase, and previous studies have shown that it is closely associated with the JAK–STAT signaling pathway [26, 27]. Therefore, we hypothesize that PTPRD may regulate the phosphorylation status of certain proteins within the JAK–STAT pathway. Confocal imaging demonstrated that in BMDMs co-induced by LPS and IFN-γ, and in IL-4-induced BMDMs, PTPRD was co-localized with P-STAT3 and P-STAT6, respectively (Fig. 4F, G). To confirm the potential interaction between PTPRD and STAT, we performed co-immunoprecipitation experiments using anti-PTPRD antibodies, which revealed significant binding between PTPRD and both STAT3 and STAT6. Furthermore, in the cardiac-derived macrophages of the 5-AzaC intervention group, the phosphorylation levels of STAT3 and STAT6 were significantly reduced (Fig. 4H–I). The excessive expression of IL-6 and IL-1β is closely associated with the continuous activation of STAT3 [28], and STAT6 can regulate the expression of fibrosis-related genes (such as CD206, Fn1, and Arg1) [29, 30], the above results suggest that the potential mechanism of 5-AzaC action may involve the reduction of CpG island methylation in the first intron region of PTPRD, thereby promoting PTPRD expression and function, which in turn inhibits the JAK-STAT pathway, ultimately leading to the suppression of the differentiation of inflammatory and pro-fibrotic macrophages.

Next, we conducted in vivo rescue experiments using the PTPRD small-molecule inhibitor 7-BIA. The results indicated that targeted inhibition of PTPRD counteracted the promotional effect of 5-AzaC on allograft survival (Fig. 4J–K). Simultaneously, we sorted infiltrating macrophages from the grafts of all three groups and performed qPCR analysis, which suggested that targeted inhibition of PTPRD counteracted the suppressive effect of 5-AzaC on the differentiation of inflammatory and pro-fibrotic macrophages (Fig. S2C).

### The nanodrug delivery system MV/PEG-LPs precisely targets DNMT1^+^ macrophages

**Fig. 5:**
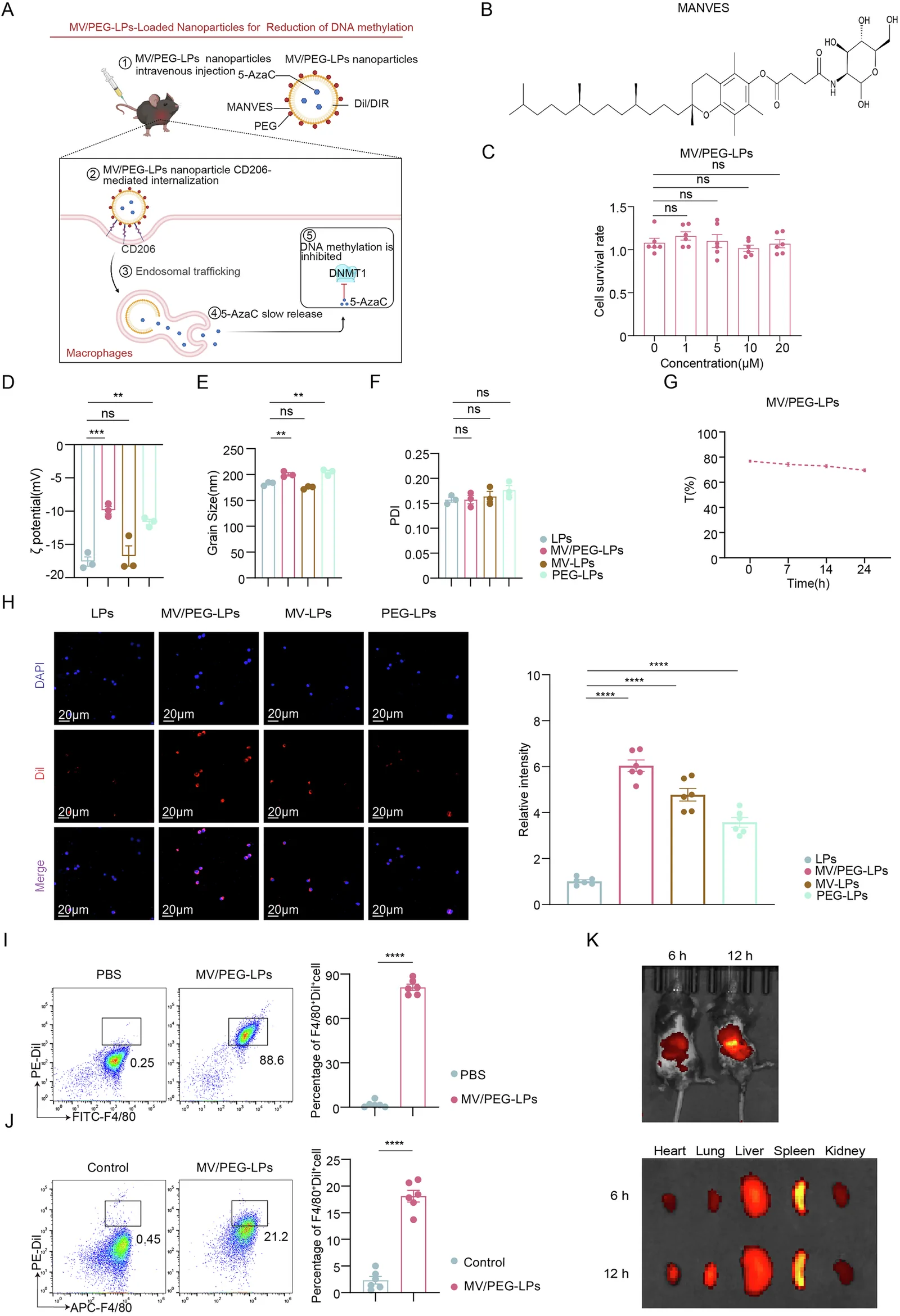
The nanodrug delivery system MV/PEG-LPs precisely targets DNMT1^+^ macrophages. **A** Schematic illustration of the structure and mechanism of action of the nanodrug delivery system MV/PEG-LPs. **B** Molecular structure of the targeting material MANVES. **C** Cell viability of RAW264.7 cells after 24 h treatment with MV/PEG-LPs containing varying concentrations of 5-AzaC (0, 1, 5, 10, 20 μM).**D**–**F** Zeta potential (mV), particle size (nm), and polydispersity index (PDI) of four nanodrug delivery systems: LPs, MV/PEG-LPs, MV-LPs, and PEG-LPs (n = 3). Unpaired Student’s *t* test. **P < 0.01; ***P < 0.001; ns = not significant. **G** Stability assessment of the nanodrug delivery system MV/PEG-LPs. Unpaired Student’s *t* test. **H** Immunofluorescence staining of BMDMs treated with LPs, MV/PEG-LPs, MV-LPs, or PEG-LPs (scale bar: 20 µm), showing Dil (red) and DAPI (blue) staining. Quantitative analysis of Dil fluorescence intensity is shown on the right (*n* = 6). Unpaired Student’s *t* test. ****P < 0.0001. **I** Flow cytometric analysis and quantification of Dil^+^ BMDMs after MV/PEG-LPs treatment (*n* = 6). Unpaired Student’s *t* test. ****P < 0.0001. **J** Flow cytometric analysis and quantification of Dil^+^ macrophages in cardiac allografts after MV^/^PEG-LPs treatment (*n* = 6). Unpaired Student’s *t* test. ****P < 0.0001. **K** Biodistribution of MV/PEG-LPs in mice at various time points, including their distribution in the heart, lung, liver, spleen, and kidney.

Given the inability of the small-molecule inhibitor 5-AzaC to specifically target macrophage DNMT1 and the significant neurotoxic side effects associated with its long-term use [31], we designed mannose-modified nanoparticles that target macrophages in vivo through CD206-mediated endocytosis, while also loading 5-AzaC to achieve targeted intervention in DNMT1 within macrophages (detailed preparation methods are described in the Materials and Methods section) (Fig. 5A). First, MANVES was synthesized via HATU-mediated amidation of mannosamine with D-α-tocopherol succinate; the product structure and purity were confirmed by 1H NMR and high-resolution TOF-MS, yielding the expected compound (Fig. 5B). MANVES, bearing a mannose moiety, can bind the macrophage surface receptor CD206, enabling macrophage targeting when incorporated into a nanodelivery system. Four fluorescently labeled liposomal formulations (Dil- or DiR-labeled LPs, MV/PEG-LPs, MV-LPs, and PEG-LPs) were then prepared by the reverse-evaporation method; DSPE-PEG2000 was included in selected formulations to prolong circulation half-life and enhance stability. Zeta potential, particle size, and polydispersity index (PDI) were measured for all four systems (Fig. 5D–F). MV/PEG-LPs exhibited a zeta potential closer to the cell membrane potential and a lower PDI than the other groups, suggesting enhanced propensity for macrophage uptake, and showed good stability at 4 °C (Fig. 5G). Immunofluorescence quantification of BMDMs treated with the four Dil-labeled formulations demonstrated the highest uptake for MV/PEG-LPs, leading us to select MV/PEG-LPs as the nanocarrier for 5-AzaC (Fig. 5H). RAW264.7 cells incubated overnight with MV/PEG-LPs loaded with varying concentrations of 5-AzaC (0, 1, 5, 10, 20 μM) showed no macrophage cytotoxicity (Fig. 5C). Flow cytometry further confirmed the targeting of MV/PEG-LPs to BMDMs and to macrophages within cardiac grafts (Fig. 5I–J). In vivo biodistribution was evaluated by tail-vein injection of DiR-labeled MV/PEG-LPs and subsequent whole-body imaging at 6 h and 12 h; DiR signal intensity in organs increased over the 12-hour period, with pronounced fluorescence observed in liver and spleen, as well as in the heart, supporting the macrophage-targeting properties of MV/PEG-LPs (Fig. 5K).

### MV/PEG-LPs prolong graft survival and alleviate graft fibrosis

**Fig. 6:**
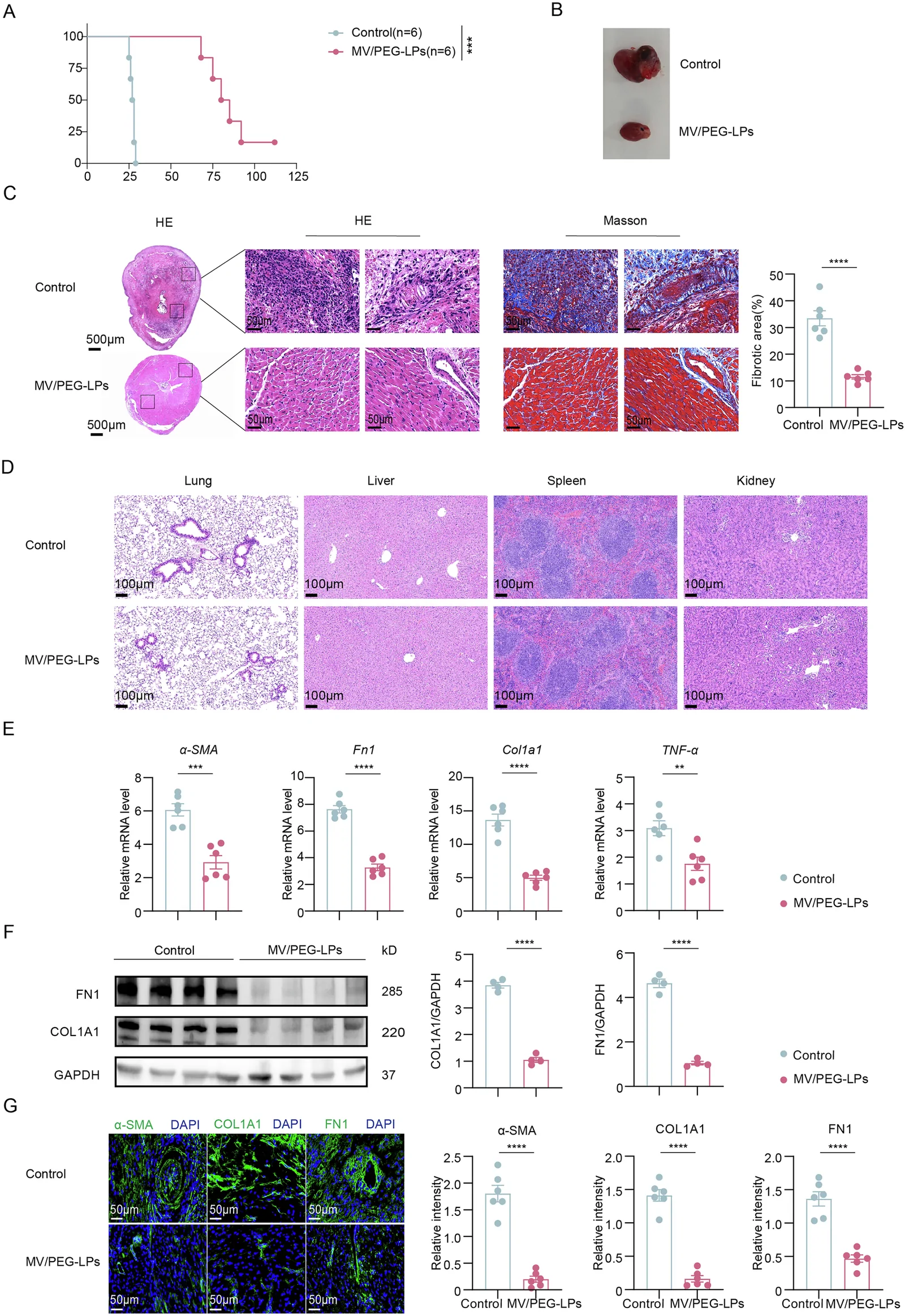
MV/PEG LPs prolong graft survival and alleviate graft fibrosis. **A** The percentage of allograft survival after transplantation (*n* = 6). Log-rank (Mantel–Cox) test. ***P < 0.001. **B** Representative images of allografts harvested on day 28 after transplantation from control and MV/PEG-LPs–treated mice. **C** Representative histological images of cardiac allografts collected on day 28 after transplantation from control and MV/PEG-LPs–treated mice, stained with H&E (scale bar: 500 or 50 µm) and Masson’s trichrome (scale bar: 50 µm). Quantitative analysis results of the fibrotic region on the right side (*n* = 6). Unpaired Student’s *t* test. ****P < 0.0001. **D** Histological assessment of major organs by H&E staining after MV/PEG-LPs treatment (scale bar: 100 µm). **E** mRNA expression levels of fibrosis-related genes and TNF-α in cardiac allografts from control and MV/PEG-LPs–treated mice (*n* = 6) at day 28 post-transplantation. Unpaired Student’s *t* test. **P < 0.01; ***P < 0.001; ****P < 0.0001. **F** Western blot analysis showing the expression levels of FN1 and COL1A1 in grafts on day 28 after transplantation, with GAPDH as the internal control. Quantification of FN1 and COL1A1 expression is shown on the right (*n* = 4). Unpaired Student’s *t* test. ****P < 0.0001. **G** Immunofluorescence analysis of allograft sections from control and MV/PEG-LPs–treated mice on day 28 after transplantation (scale bar: 50 µm), showing α-SMA, COL1A1, and FN1 (green) with DAPI nuclear staining (blue). Quantitative analysis of α-SMA, COL1A1, and FN1 fluorescence intensity is shown on the right (*n* = 6). Unpaired Student’s *t* test. ****P < 0.0001.

Utilizing the chronic rejection model, we found that mice treated with MV/PEG-LPs retained BALB/c heart allografts for a longer duration compared to PBS-treated controls (Fig. 6A). Compared to control mice, the heart allografts in MV/PEG-LPs-treated mice were significantly smaller in volume (Fig. 6B), indicating markedly reduced levels of fibrosis and immune cell infiltration in the intervention group. HE and Masson’s trichrome staining analyses revealed that, compared to control heart allografts, the MV/PEG-LPs-treated allografts exhibited only minimal fibrosis and vasculopathy (Fig. 6C). To investigate the toxicity of MV/PEG-LPs in various tissues, we used H&E staining to examine vital organs for any signs of toxicity (Fig. 6D). As shown in the figure, the MV/PEG-LPs treatment group did not exhibit any identifiable signs of toxicity, highlighting the favorable safety profile of this therapeutic approach (Fig. 6D). Next, we sorted infiltrating macrophages from the heart allografts of both groups and performed qPCR analysis, which indicated a significant reduction in the expression levels of inflammation-related and pro-fibrotic genes in macrophages from the MV/PEG-LPs intervention group (Fig. 6E). This suggests that targeting DNMT1 in macrophages inhibits the differentiation of inflammatory and pro-fibrotic macrophages, thereby suppressing the fibrosis process and promoting graft survival.

**Fig. 7:**
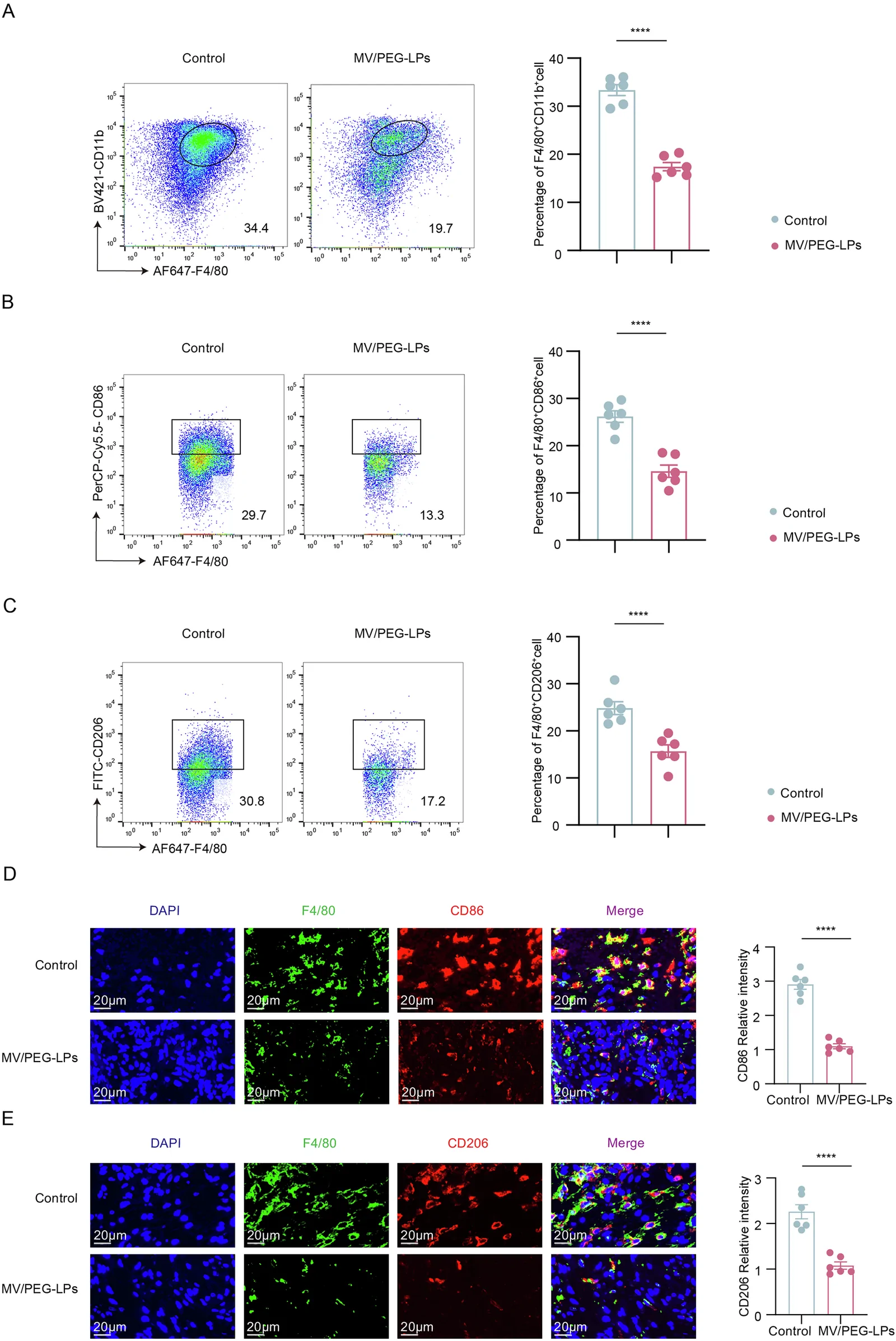
MVPEG/LPs inhibitor 5-AzaC suppresses the differentiation of inflammatory and pro-fibrotic phenotype macrophages. **A**–**C** Flow cytometric analysis and quantification of total macrophages, CD86⁺ macrophages and CD206⁺ macrophages in cardiac allografts (*n* = 6). Unpaired Student’s *t* test. ****P < 0.0001. **D** Immunofluorescence staining of F4/80 (green), CD86(red) and DAPI (blue) in cardiac allograft sections (scale bar: 20 µm), with quantitative analysis shown on the right (*n* = 6). Unpaired Student’s *t* test. ****P < 0.0001. **E** Immunofluorescence staining of F4/80 (green), CD206(red) and DAPI (blue) in cardiac allograft sections (scale bar: 100 µm), with quantitative analysis shown on the right (*n* = 6). Unpaired Student’s *t* test. ****P < 0.0001.

Furthermore, we assessed the expression of Fn1, Col1a1, and α-SMA using immunoblotting and immunofluorescence assays. The expression levels of Fn1, Col1a1, and α-SMA were significantly downregulated in the MV/PEG-LPs-treated mice (Fig. 6F, G). Thus, targeting Dnmt1 in macrophages suppresses fibrosis in chronic allograft rejection. Meanwhile, flow cytometry and immunofluorescence analyses revealed that MVPEG/LPs significantly inhibit the differentiation of macrophages into pro-inflammatory and pro-fibrotic phenotypes within cardiac grafts (Fig. 7A–E).

## Discussion

Fibrosis is a common complication in many diseases, often resulting in high morbidity and mortality [32]. Macrophages are pivotal in modulating the graft microenvironment and in the initiation and progression of fibrosis [33]. In this study, we conducted a comprehensive analysis of fibrotic heart allografts and elucidated the pivotal role of DNMT1 in the differentiation of macrophages within these fibrotic heart allografts. By employing the DNMT1 small-molecule inhibitor 5-AzaC, we demonstrated that DNMT1 primarily inhibits graft fibrosis and extends the survival of heart allografts by suppressing the differentiation of inflammatory and pro-fibrotic macrophage subsets. Mechanistically, we utilized whole-genome bisulfite sequencing and co-immunoprecipitation experiments to confirm that targeting DNMT1 reduces the DNA methylation level at the CpG islands in the first intron of PTPRD, promoting Ptprd expression, which subsequently inhibits the activity of theSTAT3 and STAT6 pathways, thereby suppressing the differentiation of inflammatory and pro-fibrotic macrophages. Building on this, we developed a macrophage-targeted Nanoparticles, MV/PEG-LPs, to encapsulate 5-AzaC, thereby enhancing its therapeutic precision for allograft fibrosis. Collectively, our findings identify DNMT1 as a critical regulator in the differentiation of inflammatory and profibrogenic macrophages in heart allografts.

Macrophages are pivotal cells that mediate inflammation and fibrosis in fibrotic diseases, characterized by their plasticity and diversity [34]. Early events in fibrosis include inflammatory changes, such as the proliferation of cells producing extracellular matrix and the onset of mononuclear inflammatory infiltration [35]. During this early stage, macrophages are activated through pattern recognition receptors by DAMPs and pathogen-associated molecular patterns (PAMPs), leading to the production of antimicrobial agents such as reactive oxygen and nitrogen species and proteases, which help eliminate invading pathogens [36]. Macrophages also produce a variety of inflammatory cytokines and chemokines, including TNF-α, IL-1, IL-6, IL-12/23, and C-C motif chemokine ligand (CCL)2, initiating a comprehensive inflammatory and antimicrobial response [37]. These responses exacerbate tissue damage and, if unresolved, lead to chronic inflammatory diseases accompanied by associated pathological fibrosis. As the fibrotic process progresses, these macrophages gradually evolve into tissue repair-associated macrophages, producing anti-inflammatory cytokine IL-10 and TGF-β [38], and playing a reparative role in the later stages of wound healing [39]. These macrophages exhibit a pro-fibrotic phenotype, driving a “benign” fibrotic response during tissue damage repair [40]. However, the overly repair by pro-fibrotic macrophages accelerates interstitial fibrosis, ultimately resulting in organ dysfunction [41].

Previous studies have demonstrated that macrophages participate in graft immune rejection and the progression of fibrosis-related diseases through epigenetic reprogramming [42, 43]. Here, by analyzing single-cell sequencing data from human heart fibrotic grafts, we identified that macrophages undergo epigenetic reprogramming characterized by DNA methylation, with DNMT1 as a highly variable gene, thereby promoting graft immune rejection and fibrosis progression. DNMT1 is an enzyme known for maintaining DNA methylation, which recognizes and binds to unmethylated CpG sites, transferring methyl groups to the newly synthesized DNA strand to maintain the methylated state of DNA in cells [44]. DNA methylation is typically associated with gene silencing. In this study, we found that intervention with the DNMT1 small-molecule inhibitor 5-AzaC significantly suppressed JAK-STAT pathway activation in heart graft macrophages. Subsequently, through whole-genome bisulfite sequencing, we identified that DNMT1 acts on the CpG island region of the first intron of the PTPRD gene. Targeted inhibition of DNMT1 resulted in demethylation and a significant restoration of PTPRD gene expression, indicating that PTPRD expression is regulated by DNMT1. Previous studies have shown that PTPRD is a confirmed target of the transcription factor STAT3, and loss of PTPRD function leads to abnormal phosphorylation of STAT3 [45]. As an inflammation-related transcription factor, the activation of STAT3 can significantly promote the expression and secretion of inflammatory molecules. Next, through co-immunoprecipitation experiments, we demonstrated that PTPRD not only binds to STAT3 but also has a significant binding association with STAT6. As a key transcription factor regulating tissue repair and fibrosis, the overactivation of STAT6 can promote fibrosis progression. By using a PTPRD small-molecule inhibitor, we confirmed that targeting PTPRD can significantly inhibit the differentiation of inflammatory and pro-fibrotic macrophage subsets, and this inhibitory effect may be caused by the suppression of phosphorylation levels of STAT3 and STAT6. Based on this, we infer that DNMT1 epigenetically reprograms macrophages through DNA methylation, silencing PTPRD gene expression, which in turn leads to excessive activation of the JAK-STAT pathway in macrophages, promoting the differentiation of inflammatory and pro-fibrotic macrophages, ultimately resulting in graft immune rejection and fibrosis, thereby affecting graft survival.

To avoid the neurotoxicity of 5-AzaC, we developed a novel nanocarrier delivery system. Macrophage-targeted nanodelivery platforms provide a powerful means to modulate innate immunity and have shown broad therapeutic potential in models of cancer immunotherapy, chronic inflammation, organ fibrosis and transplant rejection [46]. By delivering small-molecule drugs directly to macrophages, these systems can reprogram pathogenic macrophage phenotypes while reducing systemic exposure and toxicity [47]. Targeting strategies are broadly classified as passive or active: passive targeting exploits particle size, charge and surface hydrophilicity to promote accumulation in organs rich in macrophages or in inflamed lesions, whereas active targeting employs surface ligands such as mannose, which binds CD206, to promote uptake mediated by receptors [48]. In this study, we combined passive and active targeting features to develop MV/PEG-LPs with favorable macrophage-targeting properties. With established biocompatibility, MV/PEG-LPs suppress macrophage inflammatory activation and pro-fibrotic macrophage differentiation, thereby inhibiting fibrosis and prolonging graft survival.

It is undeniable that our study has certain limitations. In this study, the origin of DNMT1^+^ macrophages (i.e., the factors driving their differentiation) was not elucidated. Given the complex characteristics of macrophages within heart allografts, we only performed RNA sequencing on tissue-infiltrating macrophages isolated in vivo, without employing single-cell sequencing to precisely analyze the phenotypes of pro-inflammatory and pro-fibrotic macrophages. However, we utilized a series of in vitro assays to demonstrate that targeting DNMT1 can suppress the differentiation of inflammatory and pro-fibrotic macrophage subsets. The liver and spleen, as primary sinks for systemically administered nanoparticles, sequester a portion of particles; while this can be exploited to target resident macrophages, it also reduces delivery efficiency to other target tissues. Reducing off‑target uptake by non‑target organs will be the focus of our future investigations.

In conclusion, our study establishes a novel connection between DNA methylation and graft fibrosis. And we demonstrate that targeting macrophage DNMT1 can inhibit the differentiation of pro-inflammatory and pro-fibrotic macrophages through the PTPRD-STAT3/6 axis, thus suppressing graft fibrosis progression.

## Material and methods

### Mice

All C57BL/6J and BALB/c mice were purchased from Beijing Vital River Laboratory Animal Technology Co. Ltd. All animals were housed in the specific pathogen-free (SPF) animal facility of Tongji Hospital under controlled conditions (22 °C, 50% humidity, 12-hour light/dark cycle starting at 7:00 a.m.). The care and experimental procedures involving these mice were approved by the Animal Care and Use Committee of Huazhong University of Science and Technology. The Animal Care and Use Committee of Tongji Hospital approved all procedures involving the mice (TJ-22520230727).

### Animal model

In this study, an intra-abdominal heterotopic heart transplantation model was established. Donor hearts were obtained from BALB/c mice and transplanted into the abdominal cavity of recipient mice. During the transplantation procedure, the ascending aorta and the end of the pulmonary artery of the donor heart were anastomosed end-to-side to the recipient’s abdominal aorta and inferior vena cava, respectively. To establish a chronic rejection model, recipient mice received intraperitoneal injections of CTLA4-Ig (250 μg per dose) one day before and two days after transplantation. In addition, 5-Azacytidine was administered intraperitoneally at a dose of 5 mg/kg every other day; 7-BIA was given as a single intraperitoneal injection at a dose of 10 mg/kg; and PEG/MV-LPs were injected via the tail vein at a dose of 10 mg/kg every three days.

### Cell culture

Bone marrow was harvested from the femurs and tibias of mice and suspended in PBS. After red blood cell lysis, the bone marrow cells were transferred into DMEM medium supplemented with 10% fetal bovine serum, 1% penicillin/streptomycin, and 20 ng/mL macrophage colony-stimulating factor (M-CSF). The cells were cultured in dishes at 37 °C in a 5% CO₂ atmosphere, with the medium changed every two days. Mature macrophages were obtained after seven days of induction, and subsequently co-cultured with 10 ng/mL LPS combined with 10 ng/mL IFN-γ or 20 ng/mL IL-4 for the indicated duration according to experimental requirements. RAW264.7 cells were obtained from the China Center for Type Culture Collection. RAW264.7 cells were cultured in DMEM supplemented with 10% FBS, 100 U/mL penicillin, and 100 μg/mL streptomycin at 37 °C in 5% CO2.

### Cardiac immune cell sorting

Heart tissues harvested from mice 28 days after transplantation were minced and digested in cold Hank’s balanced salt solution (HBSS) containing collagenase II at 37 °C for 20 min. The resulting cell suspensions were gently triturated and filtered through a 70 μm cell strainer to remove residual tissue debris. After washing, the filtrated cells were subjected to density gradient centrifugation using 38% Percoll solution (GE Healthcare). The enriched immune cells will be subjected to subsequent staining according to the experimental requirements. The information on the required antibodies is as follows: Brilliant Violet 421™ anti-mouse/human CD11b Antibody(Clone M1/70, cat#: 101235), Alexa Fluor® 647 anti-mouse F4/80 Antibody (Clone BMB, cat#: 123122), PerCP/Cyanine5.5 anti-mouse CD86 Antibody (Clone GL-1, cat#: 105027), PE anti-mouse CD206 (MMR) Antibody (Clone C068C2, cat#: 141706), PE anti-mouse CD86 Antibody(Clone A17199A, cat#:159204), FITC anti-mouse F4/80 Antibody(Clone BM8, cat#:123107) and APC anti-mouse F4/80 Antibody(Clone BM8, cat#: 123115). Using the FACS Aria cell sorter (BD Biosciences), the live CD45^+^CD11b^high^F4/80^high^ phagocytes in the cardiac grafts were isolated. The sorted cells were used for RNA-seq, WGBS and qPCR experiments.

### siRNA transfection

BMDMs were differentiated from bone marrow cells using M-CSF. Once the cells had adhered, siRNA transfection was performed. The purchased siRNA stock solution had a concentration of 100 µM. Prior to transfection, the siRNA was diluted to 20 µM according to the manufacturer’s instructions. For transfection in 12-well plates, 2.5 µL of diluted siRNA was mixed with 12.5 µL of the transfection reagent enhancer. After thorough mixing, 5 µL of transfection reagent was added and mixed again. The mixture was incubated at room temperature for 5 min, followed by the addition of 30 µL of serum-free Opti-MEM medium. The resulting transfection mixture was then added dropwise to the cell culture medium. The plates were gently shaken to ensure even distribution and returned to the incubator; the medium was not changed during the culture period.

siRNA-Dnmt1 sequence: sense (5’-3’) GCCUUUACUUUCAACAUCAAATT; antisense (5’-3’) UUUGAUGUUGAAAGUAAAGGCTT.

### Histopathological study

Hearts collected 28 days after transplantation were fixed in 4% formaldehyde solution and subsequently embedded in paraffin. Tissue sections were subjected to routine histological analysis using hematoxylin and eosin (HE) staining. Masson’s trichrome staining was performed to evaluate the extent of tissue fibrosis.

### Immunofluorescent staining

Tissue sections or cell coverslips were blocked with 5% BSA at room temperature for 1 h and then incubated with primary antibodies overnight at 4 °C. After incubation, samples were washed with PBS and subsequently incubated with secondary antibodies at room temperature for 1 h. Before imaging, cells were counterstained with DAPI (Servicebio). All antibodies and their dilutions were used according to the manufacturers’ instructions. Images were acquired using a confocal microscope (Nikon TiE, Tokyo, Japan) and analyzed with NIS-Elements Viewer 5.21 and ImageJ software.

### ELISA test

ELISA was performed using the mouse IL-6 detection kit (item number:RM17709, ABclonal, China), TGF-β1 detection kit (item number: RK00057, ABclonal, China), IL-1β detection kit (item number: RM17860, ABclonal, China). The detection process was carried out strictly in accordance with the operation steps provided in the kit manual.

### Antibodies and reagents

Cell culture medium was purchased from Gibco. Protein A/G magnetic beads were obtained from Beyotime Biotechnology. Fetal bovine serum was purchased from Procell Life Science & Technology Co., Ltd. Recombinant mouse IL-4 was acquired from PEPROTECH, and recombinant mouse M-CSF protein was obtained from ABCLONAL. Primary antibodies included anti- STAT3 (catalog no. 124H6, CST, USA) anti-P-STAT3 (catalog no. D3A7, CST, USA), anti-P-STAT6 (catalog no. D8S9Y, CST, USA), anti-STAT6 (catalog no. ab32520, Abcam, USA), anti-PTPRD (catalog no. 27941-1-AP, Proteintech, China), and anti-DNMT1 (catalog no. 24206-1-AP, Proteintech, China). 5-Azacytidine and 7-BIA were purchased from MCE.Egg yolk lecithin PL‑100M and 1,2‑distearoyl‑sn‑glycero‑3‑phosphoethanolamine‑N- [amino (polyethylene glycol) -2000] (DSPE‑PEG2000) were purchased from A.V.T. Pharmaceutical Co., Ltd. (Shanghai, China). Cholesterol and D‑α‑tocopherol succinate were purchased from Aladdin Biochemical Technology Co., Ltd. (Shanghai, China). D‑glucosamine hydrochloride was purchased from Senrise Technology (Anhui, China). FBS and DMEM were purchased from Thermo Fisher Scientific Inc. (Waltham, USA). All other reagents were analytical grade and purchased from Sinopharm Chemical Reagent Co., Ltd. (Shanghai, China).

### Flow cytometry analysis

Cell suspensions obtained from heart tissues or bone marrow–derived macrophages (BMDMs) were incubated with the corresponding antibodies at 4 °C for 30 min. After washing, the cells were analyzed by flow cytometry, and the data were processed using FlowJo version 10.8 software.

### Reverse transcription quantitative PCR

Total RNA was extracted from cells using the RNA Rapid Extraction Kit (Fastagen), followed by reverse transcription with the PrimeScript RT Kit (Takara) to synthesize cDNA. Gene expression levels were quantified by real-time quantitative PCR (qPCR) using the SYBR Green Kit (Takara), analyzing the expression of target genes and the housekeeping gene HPRT simultaneously. The relative transcription levels of target genes were calculated as the ratio of their expression to that of HPRT. Fold changes in gene expression were determined using the ΔΔCT method with StepOne software (Applied Biosystems). The primer sequences used in this study are as follows:

Dnmt1-F: 5‘-AAGAATGGTGTTGTCTACCGAC-3‘, Dnmt1-R: 5‘-CATCCAGGTTGCTCCCCTTG-3‘;α-SMA-F: 5‘-GTCCCAGACATCAGGGAGTAA-3‘, α-SMA-R: 5‘-TCGGATACTTCAGCGTCAGGA-3‘; Fn1-F: 5‘-ATGTGGACCCCTCCTGATAGT-3‘, Fn1-R: 5‘-GCCCAGTGATTTCAGCAAAGG-3‘; Col1a1-F: 5‘-GCTCCTCTTAGGGGCCACT-3‘, Col1a1-R: 5‘-GCCCAGTGATTTCAGCAAAGG-3‘; Col3a1-F: 5‘-CTGTAACATGGAAACTGGGGAAA-3‘, Col3a1-R: 5‘-CCATAGCTGAACTGAAAACCACC-3‘;TGF-β1-F:5‘-CTCCCGTGGCTTCTAGTGC-3‘, TGF-β1-R:5‘-GCCTTAGTTTGGACAGGATCTG-3‘; CD86-F:5‘-TGTTTCCGTGGAGACGCAAG-3‘, CD86-R:5‘-TTGAGCCTTTGTAAATGGGCA-3‘; NOS2-F:5‘-GTTCTCAGCCCAACAATACAAGA-3‘, NOS2-R:5‘-GTGGACGGGTCGATGTCAC-3‘; IL-1β-F:5‘-GCAACTGTTCCTGAACTCAACT-3‘, IL-1β-R:5‘-ATCTTTTGGGGTCCGTCAACT-3‘; Arg-1-F:5‘-CTCCAAGCCAAAGTCCTTAGAG-3‘, Arg-1-R:5‘-AGGAGCTGTCATTAGGGACATC-3‘; CD206-F:5‘-CTCTGTTCAGCTATTGGACGC-3‘, CD206-R:5‘-CGGAATTTCTGGGATTCAGCTTC-3‘; Retnla-F:5‘-CCAATCCAGCTAACTATCCCTCC-3‘, Retnla-R:5‘-ACCCAGTAGCAGTCATCCCA-3‘; Ptprd-F:5‘-TGCCAGGTGGAAGTGTAAATATC-3‘, Ptprd-R:5‘-CGGCATATCATCTTCTGGTGTC-3‘; TNF-α-F:5‘-CCCTCACACTCAGATCATCTTCT -3‘, TNF-α-R:5‘-GCTACGACGTGGGCTACAG-3‘;

### Western blot analysis

Tissues and cultured cells were lysed in RIPA lysis buffer, and protein concentrations were determined using the BCA Protein Assay Kit (Biyuntian). After denaturation by boiling, protein samples were separated by sodium dodecyl sulfate–polyacrylamide gel electrophoresis (SDS-PAGE) and transferred onto PVDF membranes. The membranes were then blocked at room temperature for 1–2 h with TBST solution containing 5% non-fat milk, followed by incubation with primary antibodies at 4 °C for 12–16 h. Subsequently, the membranes were incubated with secondary antibodies at room temperature for 2 h, and protein expression was detected using an enhanced chemiluminescence method.

### Immunoprecipitation

IP lysis buffer containing protease inhibitors was added to the cell pellet to prepare protein lysates, which were then centrifuged at 12,000 g for 10 minutes. The supernatant was incubated with an anti-STAT3 antibody or anti-STAT6 antibody at 4 °C overnight. Subsequently, Protein A/G beads were added and incubated at 4 °C overnight to capture the immune complexes. The beads were washed four times with PBS and eluted with SDS loading buffer at 100 °C. The eluted proteins were then analyzed by immunoblotting to detect the target protein.

### RNA sequencing analysis

Total RNA was extracted from samples using TRIzol reagent (Invitrogen). Eukaryotic mRNA was selectively enriched by capturing poly(A)-tailed transcripts with oligo(dT)-modified magnetic beads. For samples with high rRNA content, rRNA was removed using specific probes or magnetic beads. The enriched mRNA was then reverse transcribed into single-stranded cDNA using reverse transcriptase, and the second strand was synthesized using DNA polymerase with the first cDNA strand as a template, yielding double-stranded cDNA. The resulting cDNA was subjected to end repair, poly(A) tailing, adaptor ligation, purification, and size selection using magnetic beads, followed by PCR amplification to construct sequencing libraries. After quality control, the libraries were subjected to high-throughput sequencing. Differentially expressed transcripts were identified by comparing gene expression profiles among samples, using the criteria of fold change (FC) ≥ 2 or ≤−2 and *P* < 0.05 for significant differences.

### WGBS analysis

Three biological replicates of cDNA samples were prepared for each group. First, sample DNA was subjected to bisulfite treatment, during which unmethylated cytosines (C) were converted to uracils (U), while methylated cytosines remained unchanged. After PCR amplification, uracils were transcribed into thymines (T), allowing the distinction between originally methylated and unmethylated cytosines. High-throughput sequencing was then performed on the Illumina NovaSeq platform to obtain raw data. Sequencing data underwent quality control using FastQC software (version 0.11.5), followed by data filtering with Trimmomatic (version 0.36) using the following parameters: ILLUMINACLIP: adapt. fa: 2:30:10 LEADING:3 TRAILING:3 SLIDINGWINDOW: 5:20 MINLEN:46. After filtering, 10 bp were trimmed from the 5′ end of R1 reads and 10 bp from the 3′ end of R2 reads. The processed data were subjected to a second quality control to obtain high-quality clean reads.Clean reads were then aligned to the reference genome using Bismark (version 0.23.1). The methylation proportions of different types of cytosine bases were calculated using cgmaptools (version 0.1.1). Differentially methylated region (DMR) analysis was also conducted with cgmaptools (version 0.1.1). Screening criteria were defined as follows: regions containing at least five cytosine sites with sequencing depth > 5×, a total region length ≤ 1000 bp, and a minimum distance of ≥200 bp between adjacent regions (<1000 bp each). DMRs between samples were identified based on differences in methylation, with thresholds of *P* ≤ 0.05 and methylation level difference (ΔmC) ≥ 0.1. Sequencing service was provided by Bioyi Biotechnology Co., Ltd. Wuhan,China.

### MANVES synthesis

MANVES was synthesized by HATU‑catalyzed amide bond formation between mannosamine and D‑α‑tocopherol succinate. D‑α‑tocopherol succinate (265.4 mg, 0.5 mmol) was dissolved in 4 mL N, N‑dimethylformamide (DMF), followed by sequential addition of N, N‑diisopropylethylamine (DIPEA, 350 μL, 1.25 mmol) and HATU (380.3 mg, 1 mmol). The mixture was stirred under nitrogen for 10 min to fully activate the carboxyl group. D‑mannosamine hydrochloride (118.6 mg, 0.55 mmol) was then added, and the reaction was allowed to proceed at room temperature for 2 h before being quenched with 40 mL distilled water. The mixture was extracted with ethyl acetate; the combined organic layers were dried over anhydrous Na2SO4 and concentrated under reduced pressure. The crude product was purified by preparative TLC using DCM/MeOH (10:1) as the eluent. Product structure and purity were verified by 1H NMR and high‑resolution time‑of‑flight mass spectrometry (TOF‑MS; Bruker, Germany).

### Preparation of liposomes

Liposomes were prepared using the reverse evaporation method. Ethanol solutions of lecithin PL‑100 M, cholesterol, DSPE‑PEG2000, and MANVES were mixed at a molar ratio of 60:30:3:7, and the organic solvent was removed by rotary evaporation. Then, 3 mL isopropanol was added to dissolve the residue, and the vial was sealed with Parafilm. The aqueous phase was prepared by dissolving 5‑AzaC in phosphate buffer (12 mM phosphate and 257 mM Glu, pH 7.42). Under ice‑bath sonication, the aqueous phase was slowly injected into the organic phase at a ratio of 6:1 (organic phase:aqueous phase) using a syringe to form a uniform water-in-oil (w/o) emulsion, followed by 3 min sonication in an ice bath. Isopropanol was then removed by rotary evaporation at room temperature. The resulting liposomes were sonicated in an ice bath for 3 min and filtered through a 0.22 μm PES syringe filter. Preparation methods for LPs (PL‑100 M: cholesterol = 63:30), PEG‑LPs (PL‑100 M: cholesterol: DSPE‑PEG2000 = 60:30:3), and MV‑LPs (PL‑100 M: cholesterol:MANVES = 63:30:7) were as previously described. Dil- or DiR-labeled liposomes were prepared similarly by mixing 1% Dil or 1.5% DiR (molar ratio) into the ethanol solution of lipid membranes before synthesis.

### In vitro stability and characterization of liposomes

The prepared liposome formulation was divided into 12 equal aliquots and stored at 4 °C in 2 mL glass vials. One aliquot was taken at day 0, 7, 14, and 24, respectively, and the absorbance at 500 nm was measured to calculate transmittance T (%); triplicate measurements (*n* = 3) were performed at each time point (the 12 aliquots provided triplicates for the four time points). Particle size, polydispersity index (PDI), and zeta potential of the liposomes were measured using a Malvern Zetasizer. Morphology was examined by transmission electron microscopy (Tecnai G2 20, FEI, Holland). Briefly, TEM samples were prepared by dropping liposome suspension onto a copper grid, allowing it to air‑dry, and staining with 2% (w/v) phosphotungstic acid for 1 min.

### CCK8

The prepared liposome formulation was divided into 12 equal portions. RAW264.7 cells were seeded in a 96‑well plate at 1 × 10^3^ cells per well and cultured overnight. Cells were then treated with 100 μL of filter‑sterilized (0.22 μm) medium containing liposome formulations with varying concentrations of 5‑AzaC (0, 1, 5, 10, and 20 μM); cell‑free blank wells received DMEM as a control. After incubation at 37 °C for 24 h, the medium was replaced with medium containing CCK‑8 reagent (10 μL per well) and incubated in the dark at 37 °C for 2 h. Absorbance at 450 nm was measured using a microplate reader, and cell viability was calculated.

### Statistical analysis

Data comparisons between groups were performed using GraphPad Prism software (version 8.0). Differences between two groups were assessed using the student’s *t* test. Survival curves were compared using the log-rank (Mantel–Cox) test. All data are presented as mean ± standard error of the mean (SEM), and differences were considered statistically significant when *P* < 0.05.

### Statistics and general methods

Sample size was not estimated using formal statistical power analysis. The number of animals (or replicates) was chosen based on previous similar experiments and practical considerations, consistent with standard practice in the field. Animals (or samples) were randomly assigned to experimental groups using a random number generator. Randomization was performed prior to treatment. Cell culture wells were randomly assigned to different stimulation conditions using a computer-generated randomization list. The experimenter was blinded to group allocation during image acquisition and data analysis. Blinding was achieved by coding the sample identifiers so that the operator did not know which stimulation condition was being processed or analyzed. Group assignments were revealed only after the data analysis was complete.

## Supplementary information

supplement figures and figure legends

blots

## Data Availability

The data that support the findings of this study are available from the corresponding author upon reasonable request.
