# Supplementary material for: DNMT1-targeted macrophages delivering drug-loaded nanoparticles attenuate heart allograft fibrosis via PTPRD-STAT3/6 axis
Source: Cell Death Discov. 2026 Aug 7;12:437. doi: 10.1038/s41420-026-03295-5 (PMC13737141; doi:10.1038/s41420-026-03295-5)
Supplement: Supplementary file 1 [file 41420_2026_3295_MOESM1_ESM.docx]

**Supplement Figures and Figure Legends:**

**Figure S1.** **Expression of DNMT1 in immune cells within human heart tissue. related to Fig. 1.**

(A-B) Expression level of DNMT1 in immune cells of human myocardium in public databases. (C) Expression of DNMT1 gene in immune cells of human geart tissue in Control and CAV groups.

**Figure S2.** **The infiltration of immune cells in the heart graft and the expression levels of macrophage markers mRNA. related to Fig. 3 and Fig. 4.**

(A) Flow cytometry analysis gating strategy. (B) Flow cytometric analysis and quantification of T cell in cardiac allografts (n = 6). Unpaired Student’s t-test. ns = not significant. (C) mRNA expression levels of *Arg‑1*, CD206, *CD86* and *NOS2* in cardiac allografts (n = 6). Unpaired Student’s t-test. ****P < 0.0001; ns = not significant.
